# Electrical stimulation of the superior sagittal sinus suppresses A-type K^+^ currents and increases P/Q- and T-type Ca^2+^ currents in rat trigeminal ganglion neurons

**DOI:** 10.1186/s10194-019-1037-5

**Published:** 2019-08-02

**Authors:** Junping Cao, Yuan Zhang, Lei Wu, Lidong Shan, Yufang Sun, Xinghong Jiang, Jin Tao

**Affiliations:** 1 Department of Physiology and Neurobiology & Centre for Ion Channelopathy, Medical College of Soochow University, 199 Ren-Ai Road, Suzhou, 215123 People’s Republic of China; 2 Department of Geriatrics & Institute of Neuroscience, The Second Affiliated Hospital of Soochow University, Suzhou, 215004 People’s Republic of China; 3 Jiangsu Province Key Laboratory of Anesthesiology, Xuzhou Medical University, Xuzhou, 221004 People’s Republic of China; 4 Jiangsu Key Laboratory of Neuropsychiatric Diseases, Soochow University, Suzhou, 215123 People’s Republic of China

**Keywords:** Migraine, Trigeminal ganglion neurons, Neuronal excitability, A-type K^+^ channels, T-type Ca^2+^ channels

## Abstract

**Background:**

Migraine is a debilitating neurological disorder involving abnormal trigeminovascular activation and sensitization. However, the underlying cellular and molecular mechanisms remain unclear.

**Methods:**

A rat model of conscious migraine was established through the electrical stimulation (ES) of the dural mater surrounding the superior sagittal sinus. Using patch clamp recording, immunofluorescent labelling, enzyme-linked immunosorbent assays and western blot analysis, we studied the effects of ES on sensory neuronal excitability and elucidated the underlying mechanisms mediated by voltage-gated ion channels.

**Results:**

The calcitonin gene-related peptide (CGRP) level in the jugular vein blood and the number of CGRP-positive neurons in the trigeminal ganglia (TGs) were significantly increased in rats with ES-induced migraine. The application of ES increased actional potential firing in both small-sized IB_4_-negative (IB_4_^−^) and IB_4_^+^ TG neurons. No significant changes in voltage-gated Na^+^ currents were observed in the ES-treated groups. ES robustly suppressed the transient outward K^+^ current (*I*_A_) in both types of TG neurons, while the delayed rectifier K^+^ current remained unchanged. Immunoblot analysis revealed that the protein expression of Kv4.3 was significantly decreased in the ES-treated groups, while Kv1.4 remained unaffected. Interestingly, ES increased the P/Q-type and T-type Ca^2+^ currents in small-sized IB_4_^−^ TG neurons, while there were no significant changes in the IB_4_^+^ subpopulation of neurons.

**Conclusion:**

These results suggest that ES decreases the *I*_A_ in small-sized TG neurons and increases P/Q- and T-type Ca^2+^ currents in the IB_4_^−^ subpopulation of TG neurons, which might contribute to neuronal hyperexcitability in a rat model of ES-induced migraine.

## Background

Migraine is a neurological disorder accompanied by a series of symptoms, including episodic manifestation and recurrent attacks of pain [1]. However, its exact pathophysiology and the underlying molecular and cellular mechanisms are not fully understood. The activation of the trigeminovascular system causes neurogenic inflammation followed by the sensitization of the central and peripheral trigeminal systems, which appears to be one of the primary mechanisms underlying this disease [2]. Emerging evidence has shown that the activity of primary afferent neurons in the rat trigeminal ganglion (TG) that innervate the dural venous sinuses contributes greatly to migraine pathophysiology [3]. Moreover, recent studies have used calcitonin gene-related peptide (CGRP) monoclonal antibodies, which are effective but do not cross the blood-brain barrier, suggesting the peripheral regulation of migraine [4, 5]. Studies have shown that the TG is not only a transition site for sensory information from the periphery to the central nervous system but that intracellular modulatory mechanisms and intercellular signalling are capable of controlling sensory information relevant for the pathophysiology of headaches [6]. Given that the sensitization of trigeminal nociceptive neurons triggers migraine headaches, studies have suggested that some receptors and ion channels in the TGs may be useful for the treatment of migraine headaches [6–8].

Voltage-gated ion channels are the primary mediators of depolarization and can be activated by different voltages. There is great diversity of channel subtypes due to the multiple genes that encode channel α1 subunits, the coassembly of a variety of ancillary channel subunits, and alternative splicing [9]. This allows these channels to fulfil highly specialized roles in specific neuronal subtypes and at particular subcellular loci [9, 10]. While ion channels are of critical importance to neuronal function, their inappropriate expression or dysfunction gives rise to a variety of neurological disorders, including epilepsy and migraine [11]. There are many diseases related to ion channels. For instance, neuronal disorders, e.g., epilepsy, episodic ataxia, familial hemiplegic migraine, and Parkinson’s disease, may result from the dysfunction of voltage-gated Na^+^ (Nav), K^+^ (Kv) and Ca^2+^ (Cav) channels [12]. Mutations in the genes that encode these channels (e.g., CACNA1A and SCN1A) have been found to cause familial hemiplegic migraine [13]. In the trigeminovascular system, voltage-gated ion channel subunits have been identified on dural afferents, and these subunits underlie trigeminal nociceptor activation and cutaneous allodynia in migraine [14, 15]. Additionally, the activation of Nav and Cav channels results in CGRP secretion from TG neurons of rats in vitro [4], indicating the role of these channels in the initiation of migraine. Nevertheless, the alteration of these channels in the pathogenesis of migraine has not been fully validated in rat animal models of migraine.

In the present study, we established a rat model of conscious migraine by electrically stimulating the dural mater surrounding the superior sagittal sinus. Based on this model, we examined whether the electrical stimulation alters voltage-gated ion channels, including Nav, Kv and Cav channels, in small-sized TG neurons. Our findings indicate that decreased A-type K^+^ currents in small-sized TG neurons and incressed P/Q-type as well as T-type Ca^2+^ currents in the small-sized IB_4_^−^ subpopulation of TG neurons can induce neuronal hyperexcitability, which would contribute to the occurrence and development of migraine.

## Materials and methods

### Animals

Sprague-Dawley rats (male, 4–5 weeks of age) were purchased from the Experimental Animal Center of Soochow University. They were housed under a 12: 12 h light/dark cycle in a temperature- and humidity-controlled room with food and water ad libitum. The experimental procedures were approved by the Institutional Animal Care and Use Committee of Soochow University and were conducted in accordance with the ethical guidelines of the International Association for the Study of Pain for experimental pain in conscious animals.

### Surgical procedures and electrical stimulation (ES)

The surgical procedures were performed according to previous studies [16] with some modifications. Briefly, the rats were anaesthetized by an i.p. injection of 4% chloral hydrate and fixed on a stereotaxic apparatus. An incision was made sagittally along the midline, and the parietal bone was clearly exposed. Two cranial windows with 1.5 mm diameters (located 4 mm anterior and 6 mm posterior to bregma along the midline suture) were prepared by drilling into the skull to expose to the dura mater surrounding of the superior sagittal sinus. A pair of stimulating electrodes (1.2 mm in diameter) was placed on the dural surface and fixed to the skull using zinc phosphate cement. All rats received prophylactic treatment with antibiotic injections for two days following surgery. After surgery, all rats were allowed to recover for 7 days without any other treatments. On the 8th day after surgery, an electrical stimulator (Mei-Yi Inc., Nanjing, China) was connected to the electrode fixed on the heads of rats and a series of electrical stimulation was initiated. The electrical stimuli were monophasic square-wave pulses with a duration of 0.25 ms, a frequency of 6 Hz and an intensity of 3–5 V, which was the minimum required to elicit a behavioural response to pain. The rats received ES 2 h per day. The animals in the sham groups were treated similarly but did not undergo electrical stimulation. After 3 days’ ES, the rats were immediately assigned to groups for analysis by enzyme-linked immunosorbent assay, electrophysiological recording and immunostaining.

### Measurement of mechanical threshold

Cutaneous allodynia is an external sign of central sensitization in migraine. Clinical studies have shown that patients with multiple migraine attacks suffer from cephalic allodynia and extracephalic allodynia [17, 18]. Therefore, the baseline mechanical threshold of the face was measured with *von* Frey monofilaments before each infusion to determine whether repeated ES stimulation of the dura induces profound mechanical allodynia. The measurement method used in this study has been described previously [19, 20]. Briefly, facial allodynia was assessed with an ascending series of *von* Frey filaments (Ugo Basile) applied to the periorbital region of the rat on the right and left side of the face over the rostral portion of the eye. A positive response was defined as a sharp retraction of the head and either the scratching of the face with the ipsilateral forepaw or the biting of the filament. For each test, the procedure was repeated three times with an interval of at least 1 min, and the average value was recorded.

### Enzyme-linked immunosorbent assay (ELISA)

The enzyme-linked immunosorbent assay was performed as previously described [21, 22]. Briefly, 1 ml of blood was collected from the external jugular vein and stored in a prechilled Eppendorf tube containing 10% ethylenediaminetetraacetic acid (15 μl) and aprotinin (500 KIU). Subsequently, the plasma was separated by cold centrifugation (1000 rpm at 4 °C for 15 min) and stored at − 80 °C until further analysis. The level of CGRP in plasma was measured with a commercial enzyme-linked immunosorbent assay kit (Wuhan USCN Business Co., Ltd) according to the manufacturer’s instructions.

### Immunoblotting

Immunoblot analysis was conducted as described in our previous studies [23–25]. Briefly, equal amounts of proteins (25 μg) were separated by 10% SDS–PAGE and electroblotted onto PVDF membranes (Merck Millipore). The blotted **proteins were** probed with the following primary antibodies: rabbit anti-Kv4.3 (1: 500, Abcam), rabbit anti-Kv1.4 (1: 600, Abcam), and rabbit anti-GAPDH (1: 5000, Abcam). After extensive washing in TBST, the membranes were incubated with horseradish peroxidase-conjugated anti-rabbit IgG (1: 10,000, Multi Sciences). Chemiluminescent signals were generated using a SuperSignal West Pico trial kit (Pierce) and detected using a ChemiDoc XRS System (Bio-Rad Laboratories). Quantity One software (Bio-Rad Laboratories) was used for background subtraction and for the quantification of the immunoblotting data.

### Immunofluorescent staining

A standard immunohistochemistry procedure was performed as described in our previous studies [25, 26]. Briefly, after tissues were sectioned (10-μm thickness) on a cryostat (CM 1950; Leica), TG and TNC sections were blocked with 5% normal goat serum in phosphate buffer saline (PBS) plus 0.15% Triton X-100 for 1 h and then incubated overnight in a primary antibody against NeuN (neuronal marker, rabbit, 1: 500, Abcam) or calcitonin gene-related peptide (CGRP) (mouse, 1: 500, Abcam). The sections were washed with PBS and incubated in Cy3-conjugated goat anti-rabbit IgG (1: 500, Cell Signaling Technology) or Dylight488 goat anti-mouse IgG (1: 500, Multi Sciences) for 2 h at room temperature. The slices were viewed under an upright fluorescence microscope (Eclipse Ts, Nikon) and images were taken using a CCD camera (Cool Snap HQ2). Negative controls incubated with secondary antibody only did not display any positive staining (not shown).

### Detection of gene expression

Reverse transcription-PCR (RT-PCR) was performed as described in our previous reports [27, 28]. Total RNA was extracted from rat TGs using the RNeasy kit (QIAGEN) according to the supplier’s instructions. Reverse transcription (RT) was performed using SuperScript™ II (Invitrogen). The primer sequences (Table 1) were designed with the Primer 5.0 software. Control reactions without RT (−RT) were used to identify contaminants in the TG samples. The reproducibility of the results was confirmed by repeating the same samples at least twice.

**Table 1:** Primers used for RT-PCR analysis

Gene	Accession number (GenBank)	Primer sequences
Kv4.1	NM_001105748.1	sense: 5′- AGAGTATAGGGACCGCAAGA −3′ anti-sense: 5′- AATGTAGTAGGGCAGGATGG − 3’
Kv4.2	NM_031730.2	sense: 5’- CGGGTTCTTCATTGCCGTCTC − 3′ anti-sense: 5‘- TTCGTTTGTCCGCTCGTTGG − 3’
Kv4.3	NM_001270962.1	sense: 5′- GCCTCACTCAAGGTTTCACA − 3′ anti-sense: 5′- CTGTTCCTCTTATGGTGGTTA − 3’
Kv1.4	NM_012971.2	sense: 5’- GCAGTCAGTTGCCCATACCT − 3′ anti-sense: 5‘- TCTCCTCGGGACCACCTTTA − 3’
Kv3.4	NM_001122776.1	sense: 5′- TTGACCGAAATGTGACGGAGAT − 3′ anti-sense: 5′- GCCAGGGAGTAGTACATACCAAAG − 3’

### Dissociation of TG neurons

TG neurons were dissociated from Sprague-Dawley rats (male, 4–5 weeks of age) according to an established protocol [29, 30]. Briefly, the bilateral TGs were dissected out and the connective tissue was removed. After being minced into 8–10 pieces, the tissues were enzymatically digested first with 1.5 mg/ml collagenase D (Roche) for 30 min and then with 1 mg/ml trypsin (Sigma) for 20 min. After trypsin treatment, the tissue was mechanically dissociated by trituration with sterile fire-polished glass pipettes. After centrifugation, the pellet was resuspended in minimum essential medium (MEM) (Invitrogen) containing 10% foetal bovine serum (FBS*) (*HyClone, GE Healthcare Life Sciences), 2% B27 supplement *(Invitrogen)*, 1% GlutaMAX, and 1% penicillin/streptomycin (Invitrogen), and then plated onto glass coverslips coated with Matrigel. Electrophysiological recordings were performed 2–6 h after plating.

### Electrophysiology recording

Electrophysiological procedures were performed at room temperature (23 ± 1 °C) as described previously [23, 24, 26]. In this study, we sorted the adult rat TG neurons into small-sized (soma diameter < 30 μm) and medium-sized (soma diameter of 30–45 μm) neurons, and performed whole-cell recording only on the small-sized neurons, as these neurons play a pivotal role in nociceptive processing [23, 31–33]. The pipettes (World Precision Instruments) had a resistance of 3–5 MΩ when filled with a pipette solution. The series resistance was compensated by at least 75% in voltage-clamp mode. The currents were filtered at 1 kHz and recorded using a MultiClamp 700B amplifier (Molecular Devices). The current traces were corrected for linear capacitive leak with online P/6 trace subtraction. A whole-cell current clamp was used to record changes in the action potential firing of the TG neurons. For whole-cell current clamp experiments and Nav current recordings, the internal solution of the electrodes contained (in mM) 10 NaCl, 110 KCl, 2 EGTA, 25 HEPES, 0.5 Na_2_GTP, and 4 Mg-ATP and had a pH of 7.3 and an osmolarity of 295 mosmol/kgH_2_O. The bath solution contained (in mM) 2 KCl, 128 NaCl, 2 CaCl_2_, 2 MgCl_2_, 30 glucose, and 25 HEPES and had a pH of 7.4 and an osmolarity of 305 mosmol/kgH_2_O. For Kv current recordings, the bath solution contained (in mM) 150 choline-Cl, 5 KCl, 0.03 CaCl_2_, 10 HEPES, 1 MgCl_2_, and 10 D-Glucose and had a pH of 7.4 and an osmolarity of 310 mosmol/kgH_2_O. The internal solution contained (in mM) 5 EGTA, 140 KCl, 0.5 CaCl_2_, 10 HEPES, 1 MgCl_2_, and 3 MgATP and had a pH of 7.4 and an osmolarity of 305 mosmol/kgH_2_O. Cav currents were recorded with an external solution containing (in mM): 20 CsCl, 140 TEA-Cl, 10 HEPES, 5 BaCl_2_, and 25 D-Glucose with a pH 7.35 and an osmolarity 305 mosmol/kgH_2_O. The internal solution of the electrodes contained (in mM) 110 CsCl, 10 EGTA, 25 HEPES, 4 MgATP, and 0.3 Na_2_GTP and had a pH of 7.4 and an osmolarity of 295 mosmol/kgH_2_O. Alexa Fluor 594-conjugated IB_4_ (Thermo Fisher) was dissolved in 0.5 mM CaCl_2_ to generate a 1 mg/ml stock solution. At the recordings, the neurons were incubated with Alexa Fluor 594-conjugated IB_4_ (3 μg/ml) to test for IB_4_ affinity.

### Data analysis

All data are expressed as the mean ± S.E.M. Clampfit 10.2 (Molecular Devices, USA) and/or GraphPad Prism 5.0 (Prism Software) was used to plot the data. The peak current amplitudes were measured at the absolute maximum of the currents. For each neuron, the peak current was normalized to the membrane capacitance (a measure of cell surface area) to reflect the current density. Differences between the groups were statistically analysed using one-way ANOVA followed by Bonferroni test or Student’s *t*-test for only two groups. The criterion for statistical significance was *p* < 0.05.

## Results

### Decreased mechanical thresholds and increased CGRP in a rat model of migraine

**Fig. 1:**
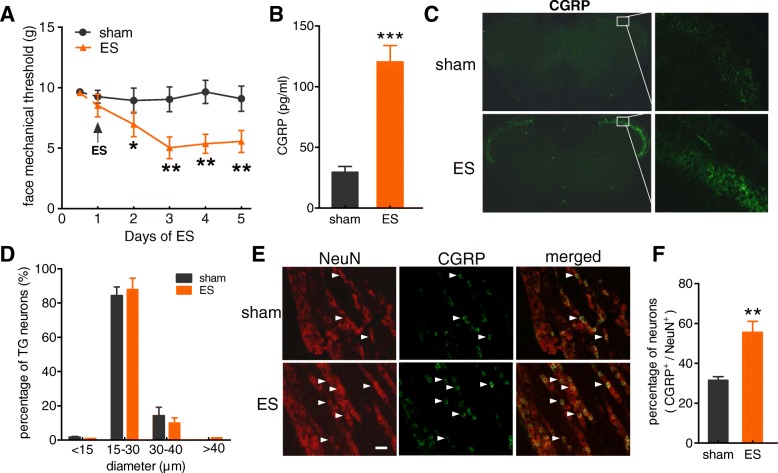
Electrical stimulation (ES) decreased the mechanical threshold and increased the CGRP level in rats. **a***,* ES induced marked mechanical face pain hypersensitivity. *p < 0.05 and **p < 0.01 vs. sham, one-way ANOVA. For animal behaviour data, *N* = at least 7 rats for each group. **b***,* ES induced a significant increase in the CGRP level in the external jugular vein of rats. ***p < 0.001 vs. sham, unpaired *t*-test. All experiments were performed in triplicate with similar results. **c***,* Immunostaining data indicating that ES significantly increased the protein expression of CGRP in trigeminal nucleus caudalis (*TNC*) sections. **d***,* Size distribution of TG neurons isolated from sham and ES-treated groups. **e***,* colocalization of NeuN (*red*) with CGRP (*green*) in rat TG sections from the sham and ES-treated groups. Arrows show the colocalization. Scale bars: 50 μm. **f***,* bar graph showing the increased percentage of CGRP-positive (CGRP^+^) TG neurons in rats with ES-induced migraine. **p < 0.01 vs. sham, unpaired *t*-test

Many animal models of migraine have been constructed through the electrical stimulation (ES) of trigeminal nerve-innervating tissues, such as the trigeminal ganglion (TG) [34], the dural arteries, the dura mater and the superior sagittal sinus [35]. In this study, we established a rat model of migraine through the ES of the dura mater surrounding the superior sagittal sinus. The baseline mechanical threshold of the face was measured by *von* Frey filaments to determine whether ES induces mechanical allodynia. There were no significant differences in the basal mechanical pain threshold of the face before ES (Fig. 1a). In addition, rats that did not undergo ES (sham) did not show any differences in mechanical pain threshold (Fig. 1a). However, the ES-treated group showed a tendency for the mechanical threshold to be decreased; furthermore, the face threshold was significantly different on day 2 (peaked at day 3) compared to that of the sham groups (Fig. 1a). Increased levels of calcitonin gene-related peptide (CGRP), but not of other neuropeptides, have been found in plasma collected from the jugular vein during migraine or cluster headache attacks [36, 37]. We therefore examined whether the level of CGRP is also increased in the rat model of ES-induced migraine and found that the application of ES significantly increased the levels of CGRP in the jugular vein blood by ~ 3-fold (increase of 300.7 ± 21.6%, Fig. 1b). CGRP is expressed in TG neurons that provide sensory innervation to the head and face and transmit nociceptive signals to the central nervous system [38, 39], suggesting its pivotal roles in trigeminovascular nociception. In our present study, immunostaining analysis indicated that ES robustly enhanced the protein expression of CGRP in the trigeminal nucleus caudalis (Fig. 1c). The size distribution of sham and ES-treated TG neurons was comparable (Fig. 1d). Further investigation of CGRP secretion in trigeminal nociceptive neurons demonstrated that CGRP was expressed mainly in small-sized rat TG neurons (soma diameter < 30 μm) (Fig. 1e). Immunofluorescence staining of rat TG sections indicated that 31.6% of total TG neurons (NeuN^+^) expressed CGRP in the sham groups, and this percentage was significantly increased to 76.3 ± 3.5% in the ES-treated groups (Figs. 1e and f).

### ES increased the neuronal excitability of rat TG neurons

**Fig. 2:**
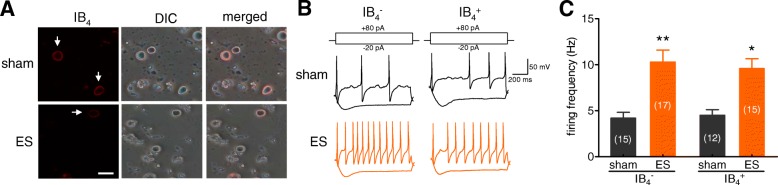
ES induced neuronal hyperexcitability in rat TG neurons **a***,* Representative images of TG neurons stained with IB_4_ in the sham and ES-treated groups. TG neurons were labelled with 3 μg/ml Alexa Fluor 594-conjugated IB_4_ for 10 min after the recordings. Scale bar = 50 μm. **b** and **c***,* Exemple traces of action potential firing (**b**) and summary data (**c**) indicating that ES significantly increased the firing frequency in both small-sized IB_4_^−^ (*left panel*) and small-sized IB_4_^+^ (*right panel*) TG neurons. Current injections of + 80 pA into the soma are shown in the top panels. *p < 0.05 and **p < 0.01 vs. sham, unpaired *t*-test

Alterations in the excitability of nociceptive sensory neurons can directly influence painful conditions such as hyperalgesia and allodynia [32, 33]. We further determined whether the application of ES affects the neuronal excitability of TG neurons. In this study, we sorted rat TG neurons according to soma diameter, grouping them into small-sized (< 30 mm) and medium-sized (30 to 45 mm) neurons, and restricted whole cell recordings to small-sized neurons because they play a critical role in the nociceptive pathway [23, 31]. IB_4_ binding is commonly used to identify non-peptidergic populations of primary afferent sensory neurons that express little or very low levels of neuropeptides [33]. Therefore, we divided the small-sized TG neurons into IB_4_-positive (IB_4_^+^) non-peptidergic neurons and IB_4_-negative (IB_4_^−^) peptidergic neurons (Fig. 2a). We found that the application of ES significantly increased the frequency of action potential firing in both small-sized IB_4_^−^ and IB_4_^+^ TG neurons (Figs. 2b and c). In addition, ES decreased the rheobase and shortened the first spike latency. Other membrane properties of neuronal excitability including the AP threshold, half-width and resting membrane potential were not significantly altered by the ES application (Table 2).

**Table 2:** Membrane properties of small-sized IB_4_^−^ and IB_4_^+^ TG neurons

		Rin (mΩ)	RMP (mV)	Rheobase (pA)	AP amplitude (mV)	AP half width (ms)	AP threshold (mV)	FSL (ms)	n
IB_4_^−^	sham	785.1 ± 73.9	−59.7 ± 5.3	85.1 ± 9.3	105.6 ± 11.8	4.7 ± 0.6	−20.3 ± 6.7	139.5 ± 28.3	15
	ES	822.5 ± 58.6	−60.8 ± 3.7	62.5 ± 10.5*	99.5 ± 7.6	4.2 ± 0.5	−20.6 ± 3.4	116.3 ± 32.3**	17
IB_4_^+^	sham	916.7 ± 73.9	−60.3 ± 7.3	89.6 ± 9.7	108.9 ± 6.5	3.8 ± 0.3	−20.5 ± 1.7	127.4 ± 19.5	12
	ES	889.5 ± 90.3	−58.5 ± 6.8	81.2 ± 9.1*	107.4 ± 6.6	3.6 ± 0.2	−21.3 ± 1.1	109.7 ± 37.3*	15

R_in_, input resistance; RMP, resting membrane potential; FSL, First spike latency; n, number of cells (*p < 0.05 and **p < 0.01 vs. sham, unpaired t-test)

### ES had no significant effects on voltage-gated Na^+^ currents

**Fig. 3:**
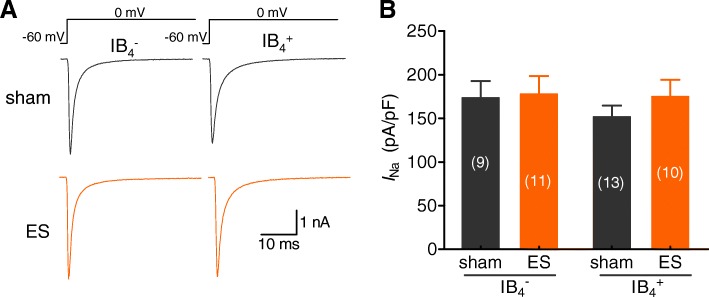
ES did not affect Nav currents. **a** and **b***,* Exemple traces of Nav currents (**a**) and summary data (**b**) indicating that ES had no significant effects on Nav current densities in either small-sized IB_4_^−^ (*left panel*) or small-sized IB_4_^+^ (*right panel*) TG neurons in the sham and ES-treated groups. Nav currents were recorded at 0 mV for 100 ms by depolarization from a − 60 mV holding potential

The abnormal excitability of primary afferent neurons may be attributable to changes in the functional characteristics and expression of voltage-gated ion channels such as Nav, Cav and Kv channels [9]. Nevertheless, it remains unclear whether these channels contribute to changes in TG neuronal excitability in a rat model of migraine. Changes in Nav currents in small-sized TG neurons were first examined in this study. Patch clamp recording analysis indicated that the application of ES had no significant effect on Nav currents in either small-sized IB_4_^−^ or IB_4_^+^ TG neurons (Figs. 3a and b), suggesting that Nav channels in small-sized TG neurons may not contribute to neuronal hyperexcitability in a rat model of ES-induced migraine.

### ES suppressed A-type K^+^ currents, but not the delayed rectifier-type K^+^ currents

**Fig. 4:**
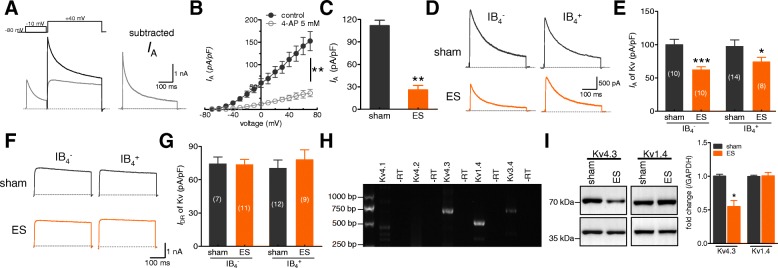
ES suppressed the *I*_A_, but did not affect the *I*_DR_ in small-sized TG neurons. **a***,* Representative current traces recorded from small-sized TG neurons. *Left:* The *I*_A_ was isolated using a two-step voltage protocol. *Right*: The *I*_A_ was obtained after the off-line subtraction of the noninactivating component. *Insets* in the top panel show the stimulation waveform, which was used for the *I*_A_ isolation indicated in all Figs. **b***,* Current-voltage (I/V) relationships of the *I*_A_ current density vs. the test potential upon treatment with 5 mM 4-AP (*n* = 6). **p < 0.01 vs. sham, one-way ANOVA. **c***,* Bar graph indicating that ES significantly decreased the *I*_A_ at + 40 mV. **p < 0.01 vs. sham, unpaired *t*-test. *D* and *E,* Representative traces (**d**) and summary data of the current density (**e**) demonstrating that ES decreased the *I*_A_ in small-sized IB_4_^−^ or IB_4_^+^ TG neurons. *p < 0.05 and ***p < 0.001 vs. sham, unpaired *t*-test. *F* and *G,* Representative current traces (**f**) and summary data (**g**) demonstrating that ES had no significant effects on the *I*_DR_ in either small-sized IB_4_^−^ or IB_4_^+^ TG neurons. **h***,* Determination of Kv1.4, Kv4 (Kv4.1, Kv4.2 and Kv4.3), and Kv3.4 mRNAs in rat TGs. No signal was detected in the reactions without RT (−RT). **i***,* Immunoblot analysis indicating the decreased protein expression of Kv4.3 in the TGs of rats with ES-induced migraine. The depicted immunoblots are representative of three different experiments. *p < 0.05 vs. sham, unpaired *t*-test

In nociceptive neurons, two main types of outward Kv currents, namely transient outward K^+^ currents (*I*_A_) and delayed rectifier K^+^ currents (*I*_DR_), have been functionally characterized [32, 40]. Therefore, we first separated the two kinetically different Kv currents in our whole-cell recordings. A large outward K^+^ current was evoked in small-sized neurons by a command potential of + 40 mV from a holding potential of − 80 mV (Fig. 4a). This typical current profile exhibited a rapid inactivation component and a subsequent sustained component. A short prepulse (150 ms) at − 10 mV inactivated the *I*_A_, leaving only the *I*_DR_. Then, the subtraction of the *I*_DR_ from the total outward current yielded the *I*_A_ (Fig. 4a). This *I*_A_ was dramatically inhibited by 5 mM 4-AP (decrease of 80.2 ± 6.5% at + 40 mV, *n* = 7; Figs. 4b and c), indicating the effective separation of the *I*_A_. The application of ES significantly decreased the current density of the *I*_A_ in both small-sized IB4^−^ and IB4^+^ TG neurons (Figs. 4d and e), while the *I*_DR_ remained unchanged in both types of TG neurons (Figs. 4f and g). The *I*_A_ in sensory neurons is thought to be mediated by Kv1.4, Kv3.4, Kv4.1, Kv4.2, and Kv4.3 channels, among which the Kv4.2 subunit is negligibly expressed [25, 41–43]. In this study, we examined the expression of Kv4 subunits (Kv4.1, Kv4.2, and Kv4.3) as well as that of Kv1.4 and Kv3.4 in rat TG tissues by RT-PCR analysis (Fig. 4h). We observed that Kv1.4 and Kv4.3 were expressed abundantly in the TG, whereas the expression levels of Kv4.1 and Kv3.4 mRNAs were relatively low. No signal of Kv4.2 mRNA was found in rat TGs (Fig. 4h). Immunoblot analysis of rat TG protein lysates further showed that the protein expression of Kv4.3, but not of Kv1.4, was significantly decreased in the ES-treated groups (Fig. 4i). These findings together indicate that Kv4.3 may contribute to the decrease in the *I*_A_ in TG neurons in a rat model of ES-induced migraine.

### ES increased P/Q-type Ca^2+^ currents in the small-sized IB_4_^−^ subpopulation of TG neurons

**Fig. 5:**
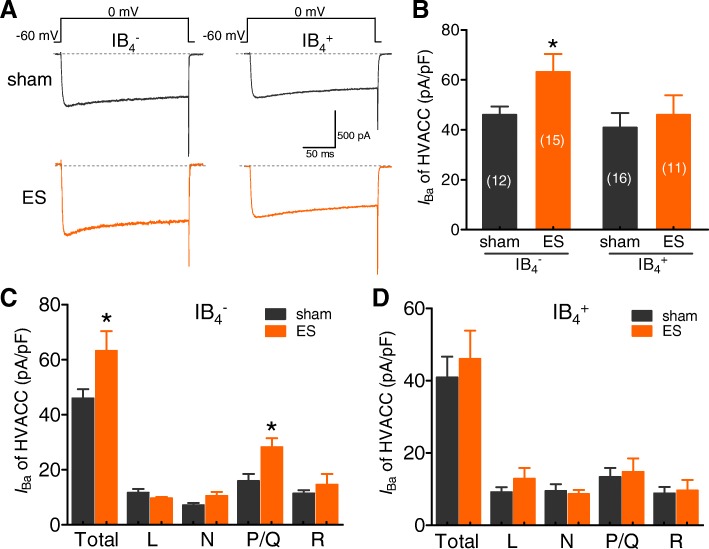
ES increased P/Q-type HVA channel currents in small-sized IB_4_^−^ subpopulation of TG neurons. **a** and **b***,* Representative current traces (**a**) and a summary of the results (**b**) demonstrating that ES significantly increased high voltage-activated (HVA) channel currents in the small-sized IB_4_^−^ subpopulations of TG neurons but not in small-sized IB_4_^+^ TG neurons. *p < 0.05 vs. sham, unpaired *t*-test. To measure the HVA channel currents, neurons were held at − 60 mV and depolarized to 0 mV (10 mV increments) for 200 ms. **c** and **d***,* The current densities of total and individual HVA channel subtypes at 0 mV in both the small-sized IB_4_^−^ (**c**) and IB_4_^+^ (**d**) subpopulations of TG neurons in the sham and ES-treated groups. The whole-cell *I*_Ba_ through individual HVA channels in small-sized TG neurons was dissected by cumulatively applying 10 μM nifedipine, 2 μM ω-conotoxin-GVIA and 0.5 μM ω-agatoxin-IVA. The remaining Cd^2+^-sensitive currents were considered R-type currents. *p < 0.05 vs. sham, unpaired *t*-test

Cav channels can be classified into high-voltage-activated (HVA) and low-voltage-activated (LVA, also known as T-type) channels [9, 44]. TG neurons express multiple types of HVA channels (L-, N-, P/Q-, and R-type) [31]. We first ask whether ES affects the total HVA currents in small-sized TG neurons. Our results showed that ES significantly increased the total HVA channel currents to a level comparable to that of small-sized IB4^−^ TG neurons in the sham groups (Figs. 5a and b). In contrast, no significant changes in total HVA channel currents were found in the small-sized IB4^+^ subpopulation of TG neurons (Figs. 5a and b). We next addressed whether the current densities from each HVA channel (L-, N-, P/Q-, and R-type) are altered as well. To address these questions, we measured whole-cell Ba^2+^ currents through each type of HVA channel. The subtype-specific HVA blockers nifedipine (10 μM), ω-conotoxin-GVIA (2 μM), and ω-agatoxin-IVA (0.5 μM) were sequentially and cumulatively introduced into the recording chamber to block L-, N-, and P/Q-type Ca^2+^ channels, respectively. The remaining Cd^2+^-sensitive (100 μM) currents were considered R-type channels. Little or no Ba^2+^ currents could be detected after Cd^2+^ application. This paradigm has been used to dissect HVA channel components in TG neurons previously [31, 45]. In the small-sized IB_4_^−^ subpopulation of TG neurons, ES significantly increased P/Q-type channel currents, while L-, N-, and R-type channel currents remained unaffected (Fig. 5c). In small-sized IB_4_^+^ TG neurons, none of the HVA channels, including L-, N-, and R-type channels, were altered by ES (Fig. 5d). These results indicate that the augmentation of P/Q-type channels in the small-sized IB_4_^−^ subpopulation may contribute strongly to the increase in total HVA channel currents in TG neurons.

### ES induced an increase in T-type currents in the small-sized IB_4_^−^ subpopulation of TG neurons

**Fig. 6:**
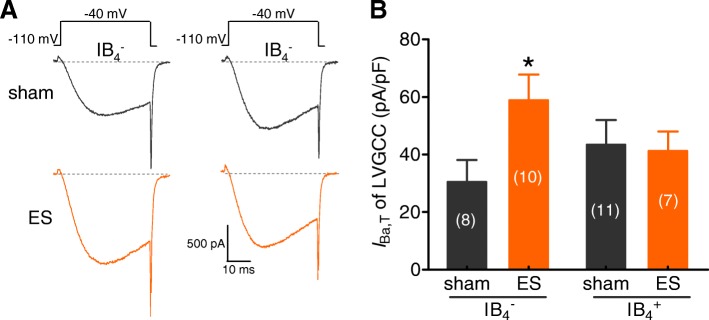
ES induced an increase in T-type LVA channel currents in the small-sized IB_4_^−^ subpopulation of TG neurons. *A* and *B,* Representative current traces (**a**) and a summary data of current density (**b**) demonstrating that ES significantly increased T-type channel currents in the small-sized IB_4_^−^ subpopulations of TG neurons but not in small-sized IB_4_^+^ TG neurons. *p < 0.05 vs. sham, unpaired *t*-test

It is well established that T-type channels regulate neuronal excitability directly by providing a depolarizing ionic current and affect synaptic transmission [10, 46]. Here we investigated the effect of ES on T-type currents in small-sized TG neurons. To record T-type currents, whole-cell Ba^2+^ currents were elicited from a holding potential of − 110 mV and stepped to − 40 mV after applying 5 μM nifedipine to block L-type channels and 0.2 μM ω-conotoxin MVIIC to block N-and P/Q-type channels (Fig. 6a). The application of 100 μM NiCl_2_ decreased the remaining inward current by approximately 83.1% (*n* = 6), confirming the isolation of T-type currents in the TG neurons. In small-sized IB_4_^−^ TG neurons, the subpopulation that is highly relevant to migraine pathophysiology [5], the application of ES significantly increased T-type currents to a level comparable to that of in the sham groups (Figs. 6a and b). Interestingly, further determination of T-type currents in the IB_4_^+^ subpopulation showed no significant changes between the sham and ES-treated groups (Fig. 6b).

## Discussion

Many migraine models involving the activation of the trigeminovascular nociceptive pathway, such as the dural artery model and the trigeminal ganglion model, have been constructed by stimulating trigeminal nerve-innervating tissues [35, 47]. However, these migraine models were established in anaesthetized animals. It is undeniable that anaesthesia has a great influence on the transmission of nociceptive information and the release of neurotransmitters. In our present study, we applied the electrical stimulation (ES) in rats that were kept conscious to better mimic the process of migraine and described the functional consequences of ES on both neuronal excitability and voltage-gated ion channel currents in small-sized TG neurons. After establishing the model of ES-induced migraine in rats, we reported for the first time that the excitability of small-sized TG neurons was significantly increased. Further investigation of channel mechanisms in peptidergic neurons and non-peptidergic TG neurons showed that the application of ES significantly decreased the *I*_A_ in both types (IB_4_^−^ and IB_4_^+^) of small-sized neurons and increased the current density of P/Q-type as well as T-type channel currents in the IB_4_^−^ subpopulation of TG neurons; however, in rats with ES-induced migraine, Nav currents as well as the *I*_DR_ were not significantly changed.

Currently, several processes, including altered activity in the hypothalamus, cortical-spreading depression, and afferent sensory input from the cranial meninges, are thought to contribute to migraine [48, 49]. The abnormal function of Nav channels may contribute to each of these processes and may thus play a role in migraine pathophysiology. It has been shown that rare forms of familial migraine are caused by mutations in *SCN1A* [50], but few studies have directly examined the role of Nav channels in migraine. Studies directly examining a connection between Nav channels and migraine, including those that have studied the efficacy of Nav blockers in both preclinical migraine models and in human migraine patients [51] have generated promising results. Although several anti-epileptic compounds, such as valproate and lamotrigine, which act through Nav channels, have been shown to be effective at reducing the frequency of migraine attacks, these drugs act on other targets, and therefore their therapeutic effect may be unrelated to their effect on Nav channels [52]. It should also be noted that use-dependent selective Nav blockers, such as phenytoin and carbamazepine, have not been documented to be efficacious against migraine attacks [53]. Therefore, studies examining the modulation of migraine by Nav blockers have led to conflicting conclusions. Although there is a large body of evidence indicating that migraine headache develops from the activation and sensitization of trigeminal sensory afferents that innervate cranial tissues, particularly the meninges and their large blood vessels [2], we found in the present study that the application of ES had little effect on the peak amplitude of Nav currents in small-sized TG neurons. These findings suggest that Nav channels may not contribute to ES-induced TG neuronal hyperexcitability in rats.

Kv channels are crucial determinants of neuronal activity throughout the nervous system. The opening of these channels facilitates a hyperpolarizing K^+^ efflux across the plasma membrane that counteracts inward ion conductance and therefore limits neuronal excitability [54]. Accumulating research has highlighted a prominent involvement of Kv channels in nociceptive processing, particularly in determining peripheral hyperexcitability in peripheral nociceptive neurons [54, 55]. For instance, sulfureted hydrogen produces mechanical pain and increases neuronal excitability in small-sized TG neurons, which is mediated by suppressing the *I*_DR_ [56]. Moreover, it has been suggested that neuronal KCNQ channels (K(V)7.2–5) represent attractive targets for the development of therapeutics for migraine, epilepsy and other neuronal hyperexcitability disorders [57]. However, in the present study, we found that the application of ES did not affect the *I*_DR_ in small-sized TG neurons but it decreased the current density of the *I*_A_ in both IB_4_^−^ and IB_4_^+^ TG neurons. Although the discrepancy requires further investigation, in the present study, the *I*_DR_ was recorded only in small-sized neurons, and it should be noted that *I*_DR_ channels such as Kv1.1 and Kv1.2 in peripheral sensory neurons are predominantly abundant in medium- and large-sized sensory neurons [40, 58]. Previous studies have suggested the differential expression profile of Kv subunits in different classes of sensory neurons. The *I*_A_ in sensory neurons is thought to be mediated by Kv1.4, Kv3.4, Kv4.1, Kv4.2, and Kv4.3 channels [25, 41, 42]. Interestingly, both strong Kv4.3 immunoreactivity in small-sized TG neurons [42] and a large number Kv1.4-positive neurons in the TGs [56] have been reported. In our present study, we observed that the TG abundantly expressed Kv1.4 and Kv4.3, while Kv4.1, Kv4.2 and Kv3.4 mRNA expression was relatively low. Further immunoblot analysis indicated that Kv4.3 might contribute to the decrease in the *I*_A_ in small-sized TG neurons. Interestingly, Kv4.3 appeared selectively in the somata of a subset of nonpeptidergic nociceptive sensory neurons [43]. The decrease of Kv4.3 expression in the present study could only account for the *I*_A_ decrease in the IB_4_^+^ TG neurons. As none of other A-type subunits down-regulated after ES, other mechanisms than transcriptional regulation, such as protein trafficking, channel phosphorylation or ubiquitination could be involved. Although further studies are needed to reveal the relationship between Kv4.3 and preventive efficacy against migraine attacks, this subtype of Kv4 has been regarded as one of the pharmacological targets for the development of new drugs against epilepsy and neuropathic pain [59]. In line with this, studies have proven that Kv4.3 in peripheral trigeminal nociceptors is effective in orofacial neuropathic pain [42]. Nevertheless, the possibility of the involvement of other potential channel targets besides A-type channels in the pathophysiology of migraine can not be ruled out. Although further investigation is clearly necessary in rats with ES-induced migraine, emerging evidence suggests that K_ATP_ channels may play a pivotal role in migraine pathogenesis and could be a potential novel therapeutic anti-migraine target [60].

Cav channels couple the depolarization of the cell-surface membrane to the entry of Ca^2+^, which triggers contraction, secretion, neurotransmission, gene expression, and other physiological responses [61]. They are encoded by ten genes, which generate the following three subfamilies: L-type (Cav1.1–1.4), P/Q-, N-, and R-type (Cav2.1–2.3), and T-type (Cav3.1–3.3) channels [61]. Although previous studies have shown that N-type and L-type channels might be involved in migraine pathophysiology [62], controversial studies have suggested that Cav channels are not involved in the generation and propagation of spreading depression in rats [63]. In this study, we found that the application of ES had little effect on both N-type and L-type HVA channel currents, while P/Q-type channel currents were significantly enhanced by the application of ES. Mutations in P/Q-type channels have been shown to result in a decrease or increase in whole-cell current density in transfected cells/neurons in FHM-1 [31, 64], and these abnormal channels may increase the activity of the trigeminal nociceptive pathway underlying migraine headache [65]. In line with our present study, previous studies have also shown that the blockade of P/Q-type channels is able to attenuate ES-induced neurogenic dural vasodilatation in rats [66]. Moreover, the blockade of the P/Q-type channels prevents presynaptic CGRP release from perivascular trigeminal sensory nerve fibres [67]. Importantly, the stimulation of CGRP release from the cell body is implicated in peripheral sensitization, while its release from terminals in the spinal cord promotes central sensitization [5], and these processes are key physiological features associated with migraine [2]. We also identified in this study that the application of ES significantly increased T-type channel currents, especially in the small-sized IB4^−^ subpopulation of TG neurons. Interestingly, a substantial percentage of IB_4_^−^ TG neurons projecting to the dura and intracranial vessels express CGRP, a neuropeptide that plays an important role in migraine pathophysiology [5]. Accumulating studies have suggested that the aberrant expression and function of T-type channels are implicated in pathological conditions, including migraine and neuropathic pain [68–70]. Although future experiments are needed to determine which Cav3 subtype(s) directly account for the hyperexcitation of small-sized IB_4_^−^ TG neurons and the relationship between CGRP and T-type channels, molecular, genetic, and functional analyses [71, 72] have illuminated that the upregulation of T-type channels in sensory neurons can facilitate and amplify pain signals originating from the periphery, making T-type channels attractive therapeutic targets for the treatment of pain conditions.

## Conclusion

Collectively, this study found that the excitability of small-sized TG neurons (both IB_4_^−^ and IB_4_^+^ neurons) was significantly increased by ES application. Further investigation of channel mechanisms in peptidergic neurons and non-peptidergic TG neurons showed that the application of ES significantly decreased the *I*_A_ in both types of neurons and increased the current density of P/Q-type as well as T-type channels in the IB_4_^−^ subpopulation of TG neurons. The currents through Nav and *I*_DR_ were not significantly changed in the rat model of ES-induced migraine. The modulation of *I*_A_ channels, T-type and P/Q-type channels may contribute to the neuronal excitability of peripheral sensory neurons and subsequently to synaptic transmission; thus, our present study may offer insights into opportunities for analgesic pharmacotherapy and provide attractive targets for migraine treatment.

## Data Availability

All data and materials generated in this study are available upon request.

## Abbreviations

4-AP: 4-aminopyridine
CGRP: Calcitonin gene related peptide
ES: Electrical stimulation
FSL: First spike latency; n: number of cells
HVA: High-voltage-activated
*I*_A_: Transient outward A-type K^+^ currents
*I*_DR_: The delayed rectifier K^+^ currents
LVA: Low-voltage-activated
R_in_: Input resistance
RMP: Resting membrane potential
SSS: Superior sagittal sinus
TG: Trigeminal ganglion
TGVS: Trigeminovascular system
TNC: Trigeminal nucleus caudalis
VGCC: Voltage-gated calcium channels
